# The Association of Knowledge, Attitudes and Access with Park Use before and after a Park-Prescription Intervention for Low-Income Families in the U.S.

**DOI:** 10.3390/ijerph17030701

**Published:** 2020-01-21

**Authors:** Nooshin Razani, Nancy K. Hills, Doug Thompson, George W. Rutherford

**Affiliations:** 1 Department of Pediatrics, UCSF Benioff Children’s Hospital Oakland, University of California at San Francisco, 5220 Claremont Ave, Oakland, CA 94608, USA; 2 Department of Epidemiology and Biostatistics, University of California, San Francisco, 675 Nelson Rising Lane, Sandler Neurosciences Center, San Francisco, CA 94158, USA; 3 San Francisco Department of Public Health, San Francisco Human Services Agency 170 Otis Street, San Francisco, CA 94103, USA; 4 Department of Epidemiology and Biostatistics, University of California, San Francisco, 550 16th Street, Box 1224, San Francisco, CA 94143-1224, USA

**Keywords:** park use, pediatrics, health, park prescriptions, behavioral theory

## Abstract

We conducted secondary data analyses of pooled data from a clinical trial that prescribed park visits to children and their caregivers in a low-income, urban setting. Data were collected at the prescribing visit (baseline) and at one and three months of follow up from 78 families. Family characteristics were identified at baseline; regression models were used to explore changes during follow up in associations of park use with knowledge, attitudes and perceived access to parks. At baseline, park users differed from non-users in demographics, knowledge of park locations, attitudes about the value of park visits, but not affinity for nature. Park users were also more likely than non-users to feel that their neighborhood was safe for children to play in. Changes in knowledge of park locations, nature affinity, and perceived access to parks were each significantly associated with increased park use by families at one and three months after the park prescription. Adjusting for age, gender, race, poverty, and US birth, increases in knowing the location of parks were associated with an increase of 0.27 weekly park visits (95% CI 0.05, 0.49; *p* = 0.016); increases in feeling a caregiver had money to visit parks were associated with 0.48 more weekly park visits (95% CI 0.28, 0.69; *p* < 0.001); increases in perceived money for park outings were associated with 0.24 increased park visits per week (95% CI 0.05, 0.42; *p* = 0.01); each unit increase in nature affinity was associated with 0.34 more weekly park visits (95% CI 0.09, 0.59; *p* = 0.007). In other words, knowing where to go, valuing nature, and having time, and money contributed to increased likelihood of visiting a park. We discuss in terms of health behavior theory how demographics, knowledge, attitudes and perceived barriers to park use can inform park prescription interventions.

## 1. Introduction

Clinicians have been increasingly interested in how to counsel their patients about time outdoors as a means to health [1,2,3,4]. One approach currently gaining popularity is “park prescriptions”, defined here as programs where clinicians prescribe or recommend park visits to encourage healthy, active living. In practice, these programs range from providers printing out maps of local parks [5], to partnerships with existing park programs [6], to health care providers leading park outings with patients [7].

The number and variety of park prescription programs continues to rise [8]. The body of experimental studies evaluating this practice has also started to build. The few experimental studies on park prescription programs to date have shown moderate adherence [9], increased physical activity [5], improved quality of life [10], and improved resilience in children who received park prescriptions [11]. A park prescription for the family demonstrated increased weekly park visits and lowered stress in parents [12]. Other nature-based therapies such as horticulture have shown biologic benefits, such as reduced pro-inflammatory cytokines seen in elderly participants of a randomized trial [13].

Park prescriptions, despite these important and preliminary experimental evaluations, are still in a formative evaluation phase; there are many opportunities for research in this emerging practice. The theoretic basis for park prescriptions is derived from both observational and experimental studies that have demonstrated a health benefit to living near parks [14,15,16] or using parks [17,18]. Evidence that exposure to nature, presumably found in many parks, promotes health across the lifespan also bolsters the practice of park prescriptions [19,20,21,22]. The biological basis for park prescriptions is further suggested by research that viewing urban versus garden scenes resulted in an increase of oxy-hemoglobin on the right area of the prefrontal cortex that indicates more stressful experience in the urban scenes [23].

While there is a large and increasing literature on the health benefits of visiting parks and nature, there is less experimental data guiding the intersection of park use and clinical medicine. Prior to the park prescription movement, motivating individuals to use parks had been investigated as a function of structural factors such as distance to parks [24], neighborhood characteristics [25], and availability of park amenities or programing [26]. This public health lens has been crucial, as access to nature and disparities in park use mirror socio-economic and health disparities [27,28]. As the field of park visits moves into a clinician’s purview, existing research seems to suggest that designing an intervention targeting the individual and family is complicated—how families prioritize outdoor activities is multi-factorial, informed by individual, interpersonal, and structural factors [29]. In the case of other complex behaviors, models for behavior change stress the importance of evaluating the perceptions, attitudes, beliefs, and outcome expectations of individuals as a crucial means to understand observed behaviors and to guide behavioral change [30,31]. Given the potential complexity informing whether patients spend time in their day outdoors for health, a clear health behavior theory for park prescriptions is needed and is currently lacking.

**Figure 1:**
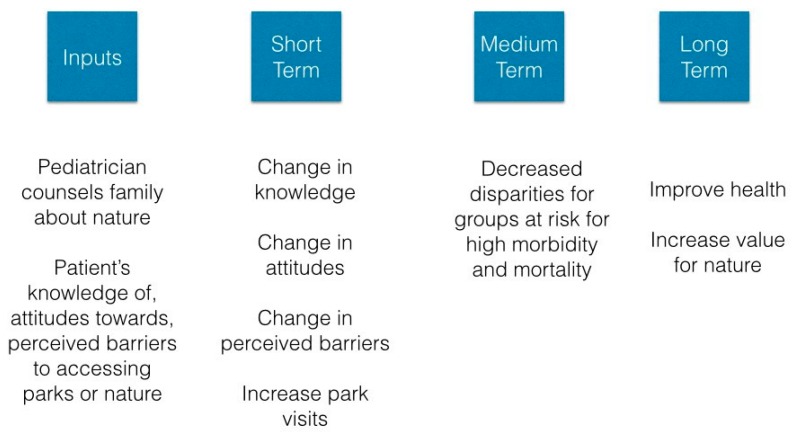
Stay Healthy in Nature Everyday logic model for an individual or family based intervention to increase park visits.

Our program is the site of an active park prescription program for low-income families. As such, we had the opportunity to extend health behavior theory to the concept of time spent in parks/nature to set the stage for effective clinical programs to encourage healthy living through time in the outdoors. We designed our park prescription program using the Precaution Adoption Process Model (PAPM) health behavior theory (detailed elsewhere and presented in simplified form in Figure 1) [7], which proposes that healthy behavior change occurs in stages. By recommending that children spend more time outdoors through a park prescription from their doctor, the program brings the role of parks in improving heath into the awareness of children and their parents and reinforces knowledge and attitudes with the aim of encouraging behavior change. Additionally, we have noted that park prescription programs assume that a park visit will impact nature exposure and affinity for those who receive it. Our prescription emphasized the health benefits of nature via park visits, and we sought to understand whether a doctor’s recommendation to visit a park is associated with nature affinity or whether it increases nature affinity over time.

In this study, we asked whether, prior to a park prescription, families who visit parks face fewer sociodemographic barriers to park use, have more a priori information about the location of parks, and attitudes that favor park use and higher affinity for nature. We then asked whether, after a park prescription, families who followed through with a park visit, or had more park visits, also had changes in their knowledge, attitudes about parks (including nature affinity), and perceived barriers to spending time in parks.

## 2. Materials and Methods

This is a secondary data analysis of pooled data from a clinical trial that prescribed park visits to children and their caregivers in a low-income, urban setting and has been described in detail elsewhere, including power and sample size calculations [7,11,12]. Patients (children ages 4–17) and one caregiver at a Federally Qualified Health Center in Oakland, California, were eligible for enrollment if they were not enrolled in a weight loss or exercise program, were able to walk and be physically active, and were available for the park outings and two follow-up visits over three months. Most of the patients were seeing their pediatrician for well-child visits, which are defined as routine doctor visits for comprehensive preventive health services; some patients were being seen for urgent care or sick visits. While a child did not have to have a health condition to be seen at the clinic, or enrolled in the study, we have shown elsewhere that the rate of chronic illness in this sample was high. Consent was obtained from each caregiver and for his/her child as a family. We gathered baseline data on knowledge, attitudes, perceived barriers and behaviors regarding park use. Each family then met with a pediatrician, who counseled them about the health benefits of nature and provided a park prescription. We provided a list of seven recommended area parks, each with high nature elements, but did not control which parks families actually visited. Families were randomized into two groups: a supported group was invited to three organized group outings to parks, and the other group was free to visit parks on their own. The supported group received weekly text messages to remind them of the benefits of nature and encourage them to visit their parks. Investigators followed up with all families, regardless of group assignment, at one and three months after the park prescription. We compensated all participating families for completing study measurements with gift cards of $20 at baseline, $20 at one month, and $20 at three months, regardless of participation in organized programs. 

All subjects gave their informed consent for inclusion before they enrolled and participated in the study. The study was conducted in accordance with the Declaration of Helsinki, and the protocol was approved by the Children’s Hospital Oakland Research Institute Institutional Review Board and registered with clinicaltrials.gov on 17 July 2015 (released November 2015) (ClinicalTrials.gov NCT02623855).

### 2.1. Outcomes

Baseline park use per week. Enrolled caregivers reported their own park visit behavior as well as the park visit behavior of their children by responding to the following questions: “How many times in the last week did you go to the park?” and “How many times did your child go to the park last week?” with responses ranging from 0 to 7 times. Self-reported park use frequency in the past week demonstrated substantial criterion validity as a measure of park use in a study of 232 adults from five U.S. locations where this question was compared over five time points to global positioning system (GPS) monitors worn by participants for three weeks. The Spearman correlation coefficient was 0.62–0.65 in comparing self-reported frequency of park visits in the last week to GPS monitoring data [32].

We described to patients that “parks” included both natural and urban parks (e.g., a neighborhood park may include a playground or sports field). We drew our definition from Bedimo-Rung’s 2005 conceptual framework which states that parks can improve health through a variety of features, which may or may not include the presence of sports fields as well as natural areas for more “passive contemplation of nature” [33]. Because this study was conducted in an urban center, we drew from others who consider local neighborhood parks with playgrounds to qualify as parks [34]. For baseline analyses, we converted park use into a binary variable categorized as no visits versus at least one visit in the past week.

Park use over time. Caregivers reported on their weekly park visits at one and three months after receiving a park prescription. The parks in which visits took place were not limited to those that we prescribed. For the purposes of this study, we used caregiver report of park visits as a proxy for family park visits. To check whether this was a fair assumption, we determined the strength of the association between caregiver and child park visits, as reported by the caregiver.

### 2.2. Predictors

Demographics. Caregivers provided information on the age of their children and on their own race, ethnicity, country of birth, and household income. Average annual income level was converted to a percent of the federal poverty level based on the U.S. Department of Health and Human Services guidelines [35].

Knowledge. Caregivers indicated how strongly they agreed with the following statement: “I have enough knowledge about the location of parks to visit them with my family.” Caregivers chose answers according to a five-point Likert scale.

Attitudes: value for parks. Caregivers indicated how strongly they agreed with the following statement: “I value visiting parks with my family,” with responses on a five-point Likert scale.

Attitudes: value for nature (nature affinity). Caregivers indicated how strongly they agreed with 15 items on the validated Love and Care for Nature (LCN) score [36] using a seven-point Likert scale that ranged from 1 (strongly disagree) to 7 (strongly agree). The final score, which we call nature affinity, is the average of the responses to the 15 items. This scale has stronger correlations to environmental stewardship behavior. Chronbach’s alpha was 0.97, and the score had strong evidence of its validity, including content, construct, and criterion-related validity [36].

Perceived access. Caregivers indicated how strongly they agreed with statements regarding feeling welcome in parks, safety, transportation, and having sufficient time, money, access to parks and access to nature. These items were chosen based on a review of the leisure science literature [37,38], which cites these as common and potential barriers to park use. Answers were given according to a five-point Likert scale. For some analyses, we collapsed the Likert scale into three values: agree, neutral and disagree—although findings were unchanged whether we used a five-point or three-point Likert scale. Caregivers additionally answered a question on how often (never, sometimes, usually or always) they felt that their child was safe in their community or neighborhood.

Other behaviors at baseline. Caregivers responded on a five-point Likert scale, with responses coded as strongly agree to strongly disagree, to the following statements: “I spent a lot of time in nature as a child,” and “I spend a lot of time in nature now.”

Knowledge, attitudes, perceived access over time. These caregiver characteristics were measured at baseline, and at one and three months after receiving a park prescription.

### 2.3. Statistical Analysis

Study analysis proceeded in two steps. First, we summarized the baseline responses to the demographic, knowledge, attitude, and access items, both for the entire sample and stratified by no vs. any park visits in the prior week. We compared baseline characteristics of the park visits versus no visits groups using Wilcoxon rank sum tests for continuous variables and chi-square or Fisher’s exact tests for categorical variables, as appropriate.

We then assessed the association between changes in reported knowledge, attitudes and perceived barriers after a park prescription and changes in park visiting behavior over the three months following a park prescription. Because our analyses included multiple measurements per person over time, we conducted regression analyses using generalized estimating equations (GEE) techniques. Regression analyses assume independence of all observations, and because observations made on the same individual over time are correlated, this assumption is violated when more than one measurement per person is included. GEE techniques are designed to account for this within-person (in addition to between-person) variability, by appropriately adjusting standard errors in the model for the additional variability [39]. We first analyzed each characteristic potentially associated with number of park visits in individual analyses that controlled for intervention group. Those variables significantly associated with the outcome at *p* ≤ 0.05 were then included in a multivariable model. We controlled for group in each model. GEE models were fit using an autoregressive correlation structure and robust variance estimators. We performed statistical analyses using Stata 14.1 (StataCorp., College Station, TX, USA).

## 3. Results

### 3.1. Baseline Findings

Seventy-eight families enrolled in the study. Seventy-two percent of the sample were living at or below 200% of the Federal Poverty Line, which is within the U.S. Department of Housing and Urban Development’s definition of a low-income home in the geographic area served by this study [40] (The U.S. Department of Health and Human Services (HHS) sets the poverty guidelines. Many programs use a percent multiple of the poverty guideline to determine eligibility for public programs such as food assistance. In the geographic area where participants in this study lived, poverty is defined by most federal programs as a 200% multiple of the Department of HHS income guideline for families). The sample was mostly non-white; 67% were African-Americans. The mean (sd) caregiver age was 38.5 (11) years (Table 1).

Baseline park use and other behaviors. Of the 78 caregivers, the majority, 49 (63%), reported having visited a park at least once during the prior week (Table 1). Twenty-one (27%) caregivers reported three or more park visits during the prior week (data not shown). At baseline, most people reported that they did not currently spend a lot of time in nature (72% disagreed) but that they did spend time in nature as children (65% agreed). Of note, caregiver and child park visits as reported by the parent were highly correlated and became more correlated over the course of the study (correlation coefficient of 0.60 at baseline, 0.72 at one month, and 0.78 at three months, *p* ≤ 0.001).

Baseline knowledge. Only a little over half of the caregivers stated that they had enough knowledge about the location of parks to visit them with their families (Table 1).

Baseline attitudes. Eighty-one percent of caregivers valued visiting parks with family members. Median (IQR) nature affinity score was 4.13 (3.5, 4.7), with a range of 1.6 to 5.1 (Table 1).

Baseline barriers to access. Most caregivers (71%) reported having a park in their neighborhood, having access to nature in their daily lives (68%), or feeling welcome in parks (83%). Money was the most commonly cited barrier with only 47% saying they had enough money to visit parks. Thirty-nine (50%) felt that their neighborhoods were usually or always safe for their children to play outside. On the other hand, 76% felt safe in parks (Table 1).

### 3.2. How Were Baseline Demographics, Knowledge, Attitudes and Barriers Associated with Park Use at Baseline?

**Figure 2:**
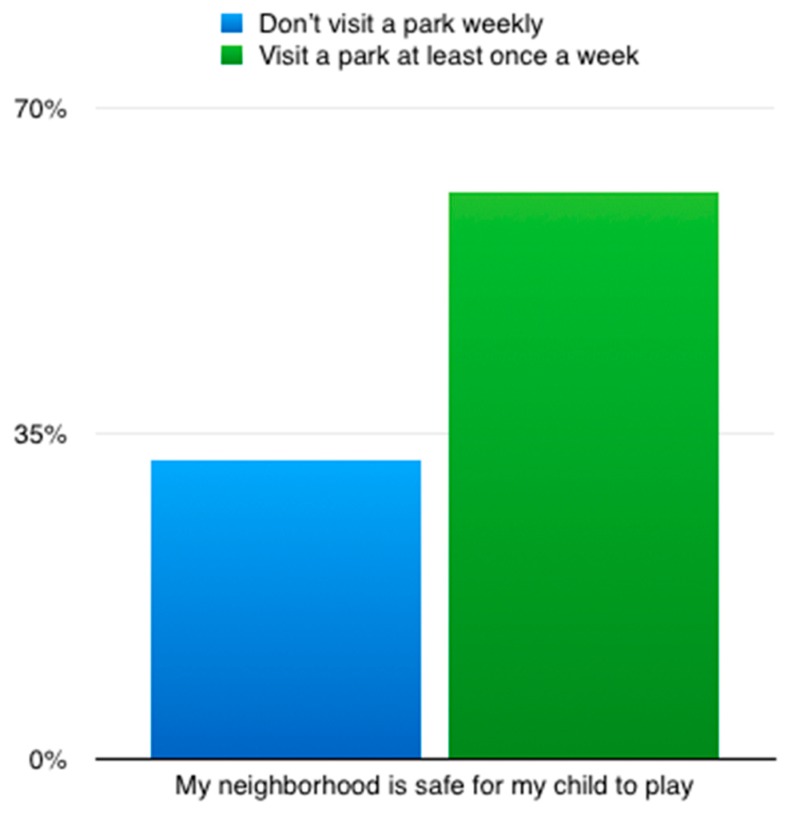
Frequency of those who have no park visits per week and those who have at least one park visit per week amongst those who believe their neighborhood is safe for children to play.

Compared to those who did not visit a park in the prior week, those who visited a park at least once were more likely to know park locations (*p* = 0.01), to value visiting parks with their families (*p* = 0.007), and to feel their neighborhoods were safe for children to play (*p* = 0.01) (Table 1 and Figure 2). Race/ethnicity of caregiver was significantly associated with having visited a park in the last week. 100% of non-Latino White participants reported visiting a park in the prior week, compared to 62% of African-Americans and 58% of Latinos. Those who visited a park at least once were also more likely to report spending a lot of time in nature now (*p* ≤ 0.001), but not more likely to report that they spent more time in nature as children (*p* = 0.87).

### 3.3. How Are Demographics, Knowledge, Attitudes and Barriers to Access Associated with Park Use as They Changed over Time after a Park Prescription?

Improvements in knowledge, attitudes, and perceived access over the course of the study were all significantly associated with increasing park visits in univariate analysis (Table 2). Once sociodemographic variables of caregiver age, gender, race, born in the USA, and percent poverty were adjusted for in a multivariable model, a more parsimonious model became clear. In the adjusted multivariable model, increasing knowledge about park locations was associated with increased visits to parks, i.e., as parents moved from one knowledge level to the next, their visits to parks increased by 0.27 [(95% CI 0.05, 0.50), *p* = 0.016] park visit per week, or about one increased park visit over three weeks. While this is a small increase, over the span of several months, it may lead to a clinically significant increase that would improve health (Table 2).

Each unit increase on the response scale for having time for visiting parks was associated with an increase of 0.48 [(95% CI 0.28, 0.69), *p* < 0.001] park visits per week. Each increase in having enough money to visit parks was associated with an increase of 0.24 [(95% CI 0.05, 0.42), *p* = 0.013] park visits per week. In other words, as the caregivers rated themselves as having more time to spend in nature, they had about one additional park visit per two weeks and as they rated themselves as having more money to spend in nature there was one additional park visit per 5 weeks (Table 2).

Finally, each additional unit of nature affinity was associated with an increase of 0.34 [(95% CI 0.09, 0.59), *p* = 0.007] park visits per week. In other words, each increase in nature affinity on a seven-point scale was associated with one additional park visit every three weeks. Age, gender, race, being born in the US, income, and group assignment did not change the final model (Table 2).

## 4. Discussion

In this study of prescribed park visits to families in a low-income, urban setting, our findings suggest that understanding perspectives, attitudes, and access to park use can be helpful in promoting healthy, active living via the outdoors. We found that at baseline, families already using parks were more likely to be white, to have prior knowledge of where to find parks, to report valuing time in parks with family, and to feel that their neighborhood was safe for their child to play; however, they were not more likely to value nature. Regardless of their baseline park use, after participants received a park prescription, park use increased as participants reported increased level of information about the location of parks, nature affinity and perceptions about time and resource availability.

This study is the first to suggest that behavioral health theory will benefit the park prescription movement. This study suggests that the same populations at risk for health inequities in chronic illness are those who may be visiting parks less at baseline. Non-white respondents and those who lacked neighborhood safety were less likely to visit parks even once a week. It is of note that this was true whether those same respondents believed they had a park in their neighborhood. This discrepancy may be explained by the fact that in the urban context, proximity barriers are not the only barriers to park use; “social access” to parks can include issues of safety, maintenance, and walkability, or issues of exclusion felt by some minorities [41]. Some studies suggest that social access is more important than proximity in understanding whether residents in a low-resource neighborhood use a park. Because these are the populations who may have the most to benefit from a park prescription program, we suggest that park prescription programs should be informed by community assessments of parks that are acceptable and accessible to families in lieu of simply recommending those which are closest.

Our study also raises the point that lower park use at baseline is not necessarily associated with lower nature affinity, or for that matter, less capacity to benefit from park prescription programs. Research showing that urban populations of color may have fears about or constraints in spending time in nature should not translate into assumptions that those populations may not desire or benefit from nature-based interventions [42]. In contrast, our study’s findings suggest that racial and ethnic minority populations can benefit from park prescriptions, even if they are not using parks at baseline. In fact, we found that the nature affinity level expressed by our cohort, even those not visiting parks at baseline, was within range of that reported in the initial Australian sample [36]. Affinity for nature also increased over time, suggesting that either the conversation with a health provider or the actual park use increased nature affinity and provided an opportunity to reinforce behavior. It is also of note that we did not find that spending time in nature as a child was associated with park use at baseline. While researchers have highlighted the importance of early childhood nature experiences, positive memories of nature and a desire to preserve the natural environment [43], our finding suggest that there are still opportunities to engage adults in park prescriptions and outdoor behavior even if they did not have these experiences in childhood.

After receiving a park prescription, shifting perceptions of barriers were associated with the changes in practice that happened after a park prescription. Increased perceived time and money to spend on park use were associated with increased park visits. It is possible that talking to a doctor about the health benefits of nature and parks changed the perceived barriers to going to parks, or that actual park use decreased the perceived financial and time barriers to being outdoors. We recommend that clinicians help patients feel comfortable getting outdoors by asking about and addressing the barriers in time, time management, or resources families may face in attempting to claim time in their week for health. In this sample, knowledge of a park to visit was associated with park use at baseline and over time. In other studies, teens and low income, urban communities of color have demonstrated willingness to travel to desirable parks even if they are outside the neighborhood [44]. Others, such as Vaughan et al. and Greer et al., have also described that efforts to promote awareness of park locations are warranted in order to reduce perceived proximity barriers to park use [45,46].

Taken as a whole, our findings suggest that it is possible to use individual targeted interventions, vis a vis a doctor’s counseling, in order to establish park use as part of a healthy, active living goal. Yet, our findings suggest that it will take more than a simple recommendation to visit parks in order to motivate families to get outdoors for health. In our study population, knowing where to find a park, feeling an affinity to nature, and having the time and resources to get outdoors were associated with patient follow through on a physician’s recommendation to get outdoors for health. The next step in research will be to define a conceptual framework, based on feedback from patients and families, that helps us understand the interplay between access and individual behavioral choices. It will be important to address the added constraint that the advent of mobile digital technology [47] places on whether families chose to spend time outdoors for health. Individual-level factors that influence outdoor time in general (including but not limited to time in parks), such as gender [48], income [49], immigration status [50], attitudes about feeling welcome [51], racism [52], family structure [53], and a person’s innate motivation to engage in outdoor activities [54], availability of social opportunities, and family rules [55,56], have each been correlated with spending time outdoors. We are interested in better understanding whether the concept of a park visit, versus time in nature, will motivate outdoor behavior. Our baseline finding that families who visited parks tended to value park visits more, but not necessarily to have high nature affinity at baseline, also speaks to the importance of better understanding whether a park or nature exposure is the concept that will motivate behavior change.

A small sample size limits this study. We did not find significant differences in income level in terms of frequency of park visits, but it is important to point out that the majority of our participants were low-income families. Our study was limited with a lack of precision in what a park is and what nature is. This limitation is true in defining the exposure across the literature in this field—keywords used to describe or classify greenspace (i.e., natural environment, schoolyard) have varied over the last decade and a half. It is difficult to compare studies when they define exposure differently or lack details of private (backyards or recreational spaces) versus public access to greenspaces. Because the study was conducted in an urban center, we were aware that patients might not have access to parks with natural elements. Our purpose in conducting this experiment was to operationalize a park prescription program in an urban clinic, with an understanding that while we referred patients to parks high in natural elements, we would not have control over which parks they visited. While we emphasized the health benefits of nature, we did not have control over the amount of nature that participants would encounter in parks they visited. A park visit is not the same as nature exposure, but it is of note that those who stated they visited parks at least once per week were more likely to state that they spend a lot of time in nature. We recommend that future studies assess the relationship between certain types of activities and specific features of different types of parks. The knowledge, attitudes, and access questions we used were not validated; this is a gap in the current literature. The predictors used in this study did not include clinical predictors. We used self-reported park use as opposed to GPS data.

## 5. Conclusions

Our study of park prescriptions, which focused on a largely low-income population in an urban center, begins to delineate a behavioral strategy to promote outdoor healthy living, and its prospective design is a major strength. It begins to set the stage to bring the topic of park and nature access, largely a public health issue until now, into the purview of clinicians. While we have in the past detailed the need to place individual-and family-based interventions in a socio-ecological model that puts park prescriptions into the context of broader social and societal issues [57], here, we present a case for addressing this public health issue from a behavioral change perspective. As trusted sources of information for parents and frontline providers of preventive health care for children, pediatricians have been on the front lines of other public health campaigns designed to influence health behaviors, even in the context of chronic illnesses with large environmental components. Understanding perspectives, attitudes, and practices related to park use can be helpful in building a health behavior strategy to promote outdoor time spent in nature as a means to increase healthy, active living in children.

## Figures and Tables

**Table 1:** Associations with zero or any weekly pre-baseline park visits and multivariable analysis of sociodemographics, attitudes, and access.

Characteristic	Total (n = 78)	(%) ^a^	No Park Visits in Prior Week (n = 29)		Any Park Visits in Prior Week (n = 49)		*p*-Value ^c^
			n	(%) ^b^	n	(%) ^b^	
**Demographics**							
Child’s age (y), median (IQR)	8 (4, 15)		29	8.6 (3.1)	49	8.9 (3.1)	0.7487
Female child	38	(49)	13	(34)	25	(66)	0.60 ^d^
Caregiver’s age, mean (SD)	73	38.5 (11)	26	36.8 (11)	47	39 (11)	0.35 ^d^
Female caregiver	68	(87)	25	(37)	43	(63)	0.55
Race/ethnicity of caregiver							0.03 *
African American	52	(67)	20	(39)	32	(62)	
Non-Latino White	8	(10)	0	(0)	8	(100)	
Latino	12	(15)	5	(42)	7	(58)	
Other ^f^	5	(6)	4	(80)	1	(20)	
Born in the US	64	(83)	26	(41)	38	(59)	0.085
% of the federal poverty level							0.07
<100%	11	(14)	5	(46)	6	(55)	
100–199	42	(54)	18	(43)	24	(57)	
200–399	12	(15)	4	(33)	8	(67)	
400% or more	9	(12)	0	(0)	9	(100)	
**Knowledge**							
I know park locations	41	(53)	9	(22)	32	(78)	0.01 *
**Attitudes**							
Nature affinity, median (IQR)	4.1	(3.5, 4.7)	3.8	(3.4, 4.2)	4.1	(3.9, 4.3)	0.18 ^e^
I value visiting parks	63	(81)	19	(30)	44	(70)	0.007 *
**Barriers to Access**							
There is a park in my neighborhood	55	(71)	18	(33)	37	(67)	0.225
I have access to nature in daily life	53	(68)	19	(36)	34	(64)	0.715
I feel welcome in parks	65	(83)	25	(39)	40	(62)	0.88
I feel safe in parks (one missing)	59	(76)	20	(34)	39	(66)	0.44
I have money to visit parks	37	(47)	12	(32)	25	(68)	0.45
I have transportation to visit parks	51	(65)	19	(37)	32	(63)	1.00
I have time to visit parks	43	(55)	12	(28)	31	(72)	0.09
My neighborhood is safe for my child to play outside	39	(50)	9	(23)	30	(77)	0.01 *
**Behaviors**							
I spend a lot of time in nature now	17	(22)	0	(0)	17	(100)	<0.001
I spent a lot of time in nature as a child	51	(65)	18	(35)	33	(65)	0.87

^a^ Column percents, ^b^ Row percents, ^c^ *p*-value calculated using Fisher’s exact test unless otherwise indicated, ^d^ *p*-value calculated using chi-square test, ^e^ *p*-value calculated using Wilcoxon rank-sum test, and ^f^ Asian, Hawaiian or Pacific Islander, American Indian or Alaskan. * Statistically significant value.

**Table 2:** Change in the number of park visits per week over baseline, one and three months compared with reported changes in knowledge, attitudes, and access after receiving a park prescription.

Characteristic	Univariate Analysis			Multivariable Analysis		
	Coeff	95% CI	*p*-Value	Coeff	95% CI	*p*-Value
Change in knowledge and attitudes						
Park in neighborhood	0.26	(0.03, 0.50)	0.03			
Access to nature	0.47	(0.24, 0.71)	<0.001			
Value visiting parks	0.53	(0.22, 0.84)	0.001			
Feel welcome in parks	0.49	(0.15, 0.82)	0.005			
Feel safe in parks	0.54	(0.30, 0.78)	<0.001			
Know park locations	0.53	(0.30, 0.76)	<0.001	0.27	(0.05, 0.50)	0.016
Have transportation to parks	0.33	(0.08, 0.58)	0.01			
Have time to visit parks	0.67	(0.43, 0.90)	<0.001	0.48	(0.28, 0.69)	<0.001
Have money to visit parks	0.43	(0.19, 0.66)	<0.001	0.24	(0.05, 0.42)	0.013
Change in nature affinity	0.65	(0.35, 0.94)	<0.001	0.34	(0.09, 0.59)	0.007
Change in neighborhood safety	0.35	(0.02, 0.70)	0.049			

We controlled for group assignment and time in the final model. Sociodemographic variables of caregiver age, gender, race, born in the USA, and percent poverty level were not significant in univariate or multivariable analysis.

## References

[1] Maller C., Townsend M., Pryor A., Brown P., Leger L.S. (2006). Healthy nature healthy people: ‘Contact with nature’ as an upstream health promotion intervention for populations. Health Promot. Int..

[2] Swinburn B.A., Walter L.G., Arroll B., Tilyard M.W., Russell D.G. (1998). The green prescription study: A randomized controlled trial of written exercise advice provided by general practitioners. Am. J. Public Health.

[3] Andrews M., Sawyer C., Frerichs L., Asheley C.S., Hoffman J., Gaskin K., Armstrong S. (2018). Feasibility of a clinic-community partnership to treat childhood obesity. Clin. Pediatr..

[4] Uijtdewilligen L., Waters C.N., Aw S., Wong M.L., Sia A., Ramiah A., Wong M., Müller-Riemenschneider F. (2019). The park prescription study: Development of a community-based physical activity intervention for a multi-ethnic Asian population. PLoS ONE.

[5] Zarr R., Cottrell L., Merrill C. (2017). Park prescription (DC Park Rx): A new strategy to combat chronic disease in children. J. Phys. Act. Health.

[6] Messiah S.E., Jiang S., Kardys J., Hansen E., Nardi M., Forster L. (2016). Reducing childhood obesity through coordinated care: Development of a park prescription program. World J. Clin. Pediatr..

[7] Razani N., Kohn M.A., Wells N.M., Thompson D., Hamilton Flores H., Rutherford G.W. (2016). Design and evaluation of a park prescription program for stress reduction and health promotion in low-income families: The stay healthy in nature everyday (SHINE) study protocol. Contemp. Clin. Trials.

[8] Seltenrich N. (2015). Just what the doctor ordered: Using parks to improve children’s health. Environ. Health Perspect..

[9] Coffey J.S., Gauderer L. (2016). When pediatric primary care providers prescribe nature engagement at a State Park, do children “fill” the prescription?. Ecopsychology.

[10] Hoffman J., Frerichs L., Story M., Jones J., Gaskin K., Apple A., Skinner A., Armstrong S. (2018). An Integrated clinic-community partnership for child obesity treatment: A randomized pilot trial. Pediatrics.

[11] Razani N., Niknam K., Wells N.M., Thompson D., Hills N.K., Kennedy G., Gilgoff R., Rutherford G.W. (2019). Clinic and park partnerships for childhood resilience: A prospective study of park prescriptions. Health Place.

[12] Razani N., Morshed S., Kohn M.A., Wells N.M., Thompson D., Alqassari M., Agodi A., Rutherford G.W. (2018). Effect of park prescriptions with and without group visits to parks on stress reduction in low-income parents: SHINE randomized trial. PLoS ONE.

[13] Ng K.S.T., Sia A., Ng M.K.W., Tan C.T.Y., Chan H.Y., Tan C.H., Rawtaer I., Feng L., Mahendran R., Larbi A. (2018). Effects of horticultural therapy on Asian older adults: A randomized controlled trial—Clinical Trial. Int. J. Environ. Res. Public Health.

[14] Wolch J., Jerrett M., Reynolds K., McConnell R., Chang R., Dahmann N., Brady K., Gilliland F., Su J.G., Berhane K. (2011). Childhood obesity and proximity to urban parks and recreational resources: A longitudinal cohort study. Health Place.

[15] Goldsby T.U., George B.J., Yeager V.A., Sen B.P., Ferdinand A., Sims D.M., Manzella B., Cockrell Skinner A., Allison D.B., Menachemi N. (2016). Urban park development and pediatric obesity rates: A quasi-experiment using electronic health record data. Int. J. Environ. Res. Public Health.

[16] Feng X., Astell-Burt T. (2017). The Relationship between neighbourhood green space and child mental wellbeing depends upon whom you ask: Multilevel evidence from 3083 children aged 12–13 years. Int. J. Environ. Res. Public Health.

[17] Cohen D.A., Ashwood J.S., Scott M.M., Overton A., Evenson K.R., Staten L.K., Porter D., McKenzie T.L., Catellier D. (2006). Public parks and physical activity among adolescent girls. Pediatrics.

[18] Giles-Corti B., Broomhall M.H., Knuiman M., Collins C., Douglas K., Ng K., Lange A., Donovan R.J. (2005). Increasing walking: How important is distance to, attractiveness, and size of public open space?. Am. J. Prev. Med..

[19] Hartig T., Mitchell R., de Vries S., Frumkin H. (2014). Nature and health. Annu. Rev. Public Health.

[20] Dadvand P., Sunyer J., Basagaña X., Ballester F., Lertxundi A., Fernández-Somoano A., Estarlich M., García-Esteban R., Mendez M.A., Nieuwenhuijsen M.J. (2012). Surrounding greenness and pregnancy outcomes in four Spanish birth cohorts. Environ. Health Perspect..

[21] Mitchell R., Popham F. (2008). Effect of exposure to natural environment on health inequalities: An observational population study. Lancet.

[22] Ulrich R.S. (1984). View through a window may influence recovery from surgery. Science.

[23] Yu J. Prefrontal cortical activation while viewing urban and garden scenes: A pilot fNIRS study. Proceedings of the 2017 39th Annual International Conference of the IEEE Engineering in Medicine and Biology Society (EMBC).

[24] Van Dyck D., Sallis J.F., Cardon G., Deforche B., Adams M.A., Geremia C., De Bourdeaudhuij I. (2013). Associations of neighborhood characteristics with active park use: An observational study in two cities in the USA and Belgium. Int. J. Health Geogr..

[25] Cohen D.A., Han B., Nagel C.J., Harnik P., McKenzie T.L., Evenson K.R., Marsh T., Williamson S., Vaughan C., Katta S. (2016). The first national study of neighborhood parks: Implications for physical activity. Am. J. Prev. Med..

[26] Dunton G.F., Almanza E., Jerrett M., Wolch J., Pentz M.A. (2014). Neighborhood park use by children: Use of accelerometry and global positioning systems. Am. J. Prev. Med..

[27] Casey J.A., James P., Cushing L., Jesdale B.M., Morello-Frosch R. (2017). Race, ethnicity, income concentration and 10-year change in urban greenness in the United States. Int. J. Environ. Res. Public Health.

[28] Wen M., Zhang X., Harris C.D., Holt J.B., Croft J.B. (2013). Spatial disparities in the distribution of parks and green spaces in the USA. Ann. Behav. Med..

[29] Godbey G.C., Caldwell L.L., Floyd M., Payne L.L. (2005). Contributions of leisure studies and recreation and park management research to the active living agenda. Am. J. Prev. Med..

[30] Glanz K., Bishop D.B. (2010). The role of behavioral science theory in development and implementation of public health interventions. Annu. Rev. Public Health.

[31] Groshong L., Stanis S.A., Kaczynski A.T., Hipp J.A., Besenyi G.M. (2017). Exploring attitudes, perceived norms, and personal agency: Insights into theory-based messages to encourage park-based physical activity in low-income urban neighborhoods. J. Phys. Act. Health.

[32] Evenson K.R., Wen F., Golinelli D., Rodriguez D.A., Cohen D.A. (2013). Measurement properties of a park use questionnaire. Environ. Behav..

[33] Bedimo-Rung A.L., Mowen A.J., Cohen D.A. (2005). The significance of parks to physical activity and public health: A conceptual model. Am. J. Prev. Med..

[34] Parks. https://www.tpl.org/parks.

[35] U.S. Federal Poverty Guidelines Used to Determine Financial Eligibility for Certain Federal Programs. https://aspe.hhs.gov/poverty-guidelines.

[36] Perkins H.E. (2010). Measuring love and care for nature. J. Environ. Psychol..

[37] Shores K., Scott D., Floyd M. (2007). Constraints to outdoor recreation: A multiple hierarchy stratification perspective. Leis. Sci..

[38] Floyd M.F., Crespo C.J., Sallis J.F. (2008). Active living research in diverse and disadvantaged communities. Am. J. Prev. Med..

[39] Zeger S.L., Liang K.Y., Albert P.S. (1988). Models for longitudinal data: A generalized estimating equation approach. Biometrics.

[40] Department of Housing Authority and Development Median Family Income Documentation System 2015. https://www.huduser.gov/portal/datasets/il/il2019/2019MedCalc.odn.

[41] Weiss C.C., Purciel M., Bader M., Quinn J.W., Lovasi G., Neckerman K.M., Rundle A.G. (2011). Reconsidering access: Park facilities and neighborhood disamenities in New York City. Urban Health.

[42] Yoshino A., Wilson J., Velazquez E.J., Johnson E., Márquez-Magaña L. (2018). Healthy parks healthy people as an upstream stress reduction strategy. Recreat. Park Tour Public Health.

[43] Wells N.M., Lekies K.S. (2006). Nature and the life course: Pathways from childhood nature experiences to adult environmentalism. Chil. Youth Environ..

[44] Edwards N., Hooper P., Knuiman M., Foster S., Giles-Corti B. (2015). Associations between park features and adolescent park use for physical activity. Int. J. Behav. Nutr. Phys. Act..

[45] Vaughan C.A., Colabianchi N., Hunter G.P., Beckman R., Dubowitz T. (2018). Park use in low-income urban neighborhoods: Who uses the parks and why?. Urban Health.

[46] Greer A.E., Castrogivanni B., Marcello R. (2017). Park use and physical activity among mostly low-to-middle income, minority parents and their children. J. Phys. Act. Health.

[47] Twenge J.M., Campbell W.K. (2018). Associations between screen time and lower psychological well-being among children and adolescents: Evidence from a population-based study. Prev. Med. Rep..

[48] Derose K.P., Han B., Williamson S., Cohen D.A. (2018). Gender disparities in park use and physical activity among residents of high-poverty neighborhoods in Los Angeles. Women’s Health Issues.

[49] Das K.V., Fan Y., French S.A. (2017). Park-use behavior and perceptions by race, hispanic origin, and immigrant status in Minneapolis, MN: Implications on park strategies for addressing health disparities. J. Immigr. Minor Health.

[50] Floyd M.F. (1999). Race, Ethnicity and Use of the National Parks System. Soc. Sci. Res. Rev..

[51] Roberts N.S., Chitewere T. (2017). Speaking of justice: Exploring ethnic minority perspectives of the Golden Gate National recreation area. Environ. Pract..

[52] Fan Y., French S.A., Das K.V. (2012). Family structure and park use among parents. Am. J. Prev. Med..

[53] Lin B.B., Fuller R.A., Bush R., Gaston K.J., Shanahan D.F. (2014). Opportunity or orientation? Who uses urban parks and why. PLoS ONE.

[54] Piccininni C., Michaelson V., Janssen I., Pickett W. (2018). Outdoor play and nature connectedness as potential correlates of internalized mental health symptoms among Canadian adolescents. Prev. Med..

[55] Cleland V., Timperio A., Salmon J., Hume C., Baur L.A., Crawford D. (2010). Predictors of time spent outdoors among children: 5-year longitudinal findings. J. Epidemiol. Community Health.

[56] Remmers T., Broeren S.M., Renders C.M., Hirasing R.A., van Grieken A., Raat H. (2014). A longitudinal study of children’s outside play using family environment and perceived physical environment as predictors. Int. J. Behav. Nutr. Phys. Act..

[57] Razani N., Stookey J., Brainin-Rodriguez L., Rutherford G.W., Chan C. (2016). Surmounting barriers to public health/park agency partnerships: Insights from a county public health department. J. Park Recreat. Ad..

